# 

**Published:** 1884-10

**Authors:** 


### CONTENTS.

ORIGINAL COMMUNICATIONS: PAGE.

I. Remarks on the Operation of Excision of Hip and Knee Joints. By Chr. Fenger, Chicago ..... 289

II. Chronic Adhesive Perimetritis. By James H. Etheridge, Chicago ..... 321

III. Life Insurance and the Medical Profession. By I. N. Danforth ..... 349

IV. Case of Uric Acid Calculus, which Appeared to Have Been Dissolved in the Pelvis of the Left Kidney by Alkaline Remedies. By Harlan W. Orton, Minneapolis, Minn ..... 3[illegible]0

EDITORIAL:

As to the Value to Physicians of the Summer Vacation ..... 362

A Case of Fatal Chloroform Narcosis ..... 363

BOOK REVIEWS:

Diseases of the Heart and Thoracic Aorta. By Byron Bramwell, M.D., F.R.C.P. ..... 366

A Treatise on the Diseases of the Eye. By J. Soelberg Wells, F.R.C.S. ..... 370

Text Book of the Diseases of the Ear and Adjacent Organs. By Dr. Adam Politzer ..... 371

The Diagnosis and Treatment of Diseases of the Ear. By Orin D. Pomeroy, M.D. ..... 372

Student's Guide to the Diseases of the Eye. By Edward Nettleship, F.R.C.S. ..... 373

SOCIETY PROCEEDINGS:

Chicago Society of Opthalmology and Otology 375

CHICAGO:

